# Micro-Organisms of the Mouth

**Published:** 1893-01

**Authors:** Jo. H. Linsley

**Affiliations:** Prof. of Pathology and Bacteriology, Medical Department, University of Vermont, Pathologist to the New York Infant Asylum, etc., Burlington, Vt.


AMERICAN JOURNAL

—OF—

DENTAL SCIENCE.

VOL. XXVI. BALTIMORE, JANUARY, 1893. NO. 9.

ARTICLE I.

### MICRO-ORGANISMS OF THE MOUTH.

BY JO. H. LINSLEY, M. D.,

Prof. of Pathology and Bacteriology, Medical Department, University of Vermont, Pathologist to the New York Infant Asylum, etc., Burlington, Vt.

Read before the Vermont State Dental Society, March, 1892.

I have many times had the misfortune to be obliged to seek the aid of members of your profession, and the treatment I have sometimes been subjected to by them, while very generally resulting in my benefit, was of a very intense and extractive nature.

I never anticipated being especially called upon to attempt an elucidation of any subject having a direct bearing on the details and operative work, incident to the practice of your profession, and even an intelligent discourse on the theme of which I speak to-day, would, but a very few short years ago, have been practically impossible and excited but little interest.

But when a representative of your society, four or five weeks ago, solicited of me a paper on "Bacteria of the Mouth," to be presented at the present session of your association, I consented to his proposition with pleasure, more especially as I had intended, shortly, to undertake some bacteriological investigations in this direction. I very much regret the limited amount of time I have had, in which to properly conduct many practical researches of some of the surprisingly numerous varieties of micro-organisms which either find their habitat in the oral cavity, or are simply resting strangers, or *loungers*, as I might say, in this locality. While I shall exhibit to you some flourishing so-called "pure cultures" of bacteria, whose ancestors were removed from carious teeth, inflamed gums, and improperly cleaned "grinders," I have, necessarily, been obliged to resort to the latest text-book on the subject, and to the various papers and articles which have recently appeared in medical and dental periodicals, both domestic and foreign, for the recognized varieties of germs infesting the mouth, their relatively respective frequency, etc.

To accomplish more than this, under the circumstances, was impossible, and the procuring of even the scanty material, which I shall present to you, exacted much more time and labor, than you might at first imagine.

It is an interesting fact in the history of bacteriology, that the first authenticated record and drawings of bacteria, were made from micro-organisms discovered in mucus from the human mouth by Leeuwenhock in 1683. From this time, until about 1860, but little progress was made in this subject, and the rapid strides, and the accumulation of important data accomplished in recent years, has been very largely due to the improvement and perfecting of optical instruments. The fortunate discovery, by Koch, and the use of artificial, solid, transparent food-media, was of scarcely secondary importance in the development of bacteriology,* by facilitating the separation, culture, and examination of germs.

*"Micro-organisms of Human Mouth."—MILLER.

A brief description of the more common culture media used for separating and growing organisms of the mouth, together with the methods employed in obtaining pure cultures will be of interest to those of you who are unfamiliar with the details of bacteriological investigations.

*Nutrient Gelatine.*—This is composed of beef juice or beef extract, to which is added from 5 per cent. to 20 per cent. of best French gelatine, 1-10 per cent. dried peptone, and 1-20 per cent. common salt. The reaction must be neutral, or very slightly alkaline. The addition of 1 per cent. to 2 per cent. of sugar improves the medium for the growth of mouth-bacteria.

This material is easily prepared,† and is largely employed, especially for plate or dish cultures. It is unfit for use in the incubator, as the gelatine is liquified at temperature above 25° C. The characteristic growth of many bacteria is better exhibited on this substance than on the following.

*Nutrient Agar-agar.*—This is prepared‡ similarly to the foregoing, only that 1 per cent. to 2 per cent. agar-agar is used, instead of the 5 per cent. to 20 per cent. of gelatine. (For detailed directions for the preparation of these media, reference to the text-books on Bacteriology is advised.)

*Boiled Potato.*—This is a very simple and valuable nutrient medium. Any sound potato can be used, excepting those which crack open, or become mealy, on boiling. The potato is carefully washed and scrubbed with a stiff brush (an ordinary nail-brush answers very well), and the "eyes," and any unsound portions, removed; it is then soaked for one hour in a 1 : 1000 bichloride of mercury solution, and finally boiled in a steam sterilizer, or cooking "steamer," for one-half or three-quarters of an hour. It is then placed in a moist chamber, or covered glass dish,

† It can be obtained already prepared bp Elmer & Amend, 205, etc., Third Ave., New York.

‡ This can also be bought of the same firm.

which has been sterilized, and in the bottom of which has been placed a piece of filter, or blotting paper, slightly moistened with a 1 : 2,000 bichloride solution.

If the proper precautions have been observed in their preparation, potatoes thus treated will remain germ free indefinitely, and can be used at any time at a moment's notice, for the planting of material from which cultures are desired. Many bacteria exhibit their most characteristic growth on this medium, and but few germs are known which refuse to exist on it.

Occasionally a micro-organism is met with which requires for its full development a different soil from any of the three media just enumerated. In such cases other substances are used, such as sterilized blood serum, starch-paste, boiled hen's egg, etc.

Liquid media are also employed in the cultivation of micro-organisms generally, as well as those from the mouth, but more especially for studying the progress and phenomena of putrefaction, fermentation, decomposition, etc., occasioned by the action of bacteria. Such media are bread juice, peptonized beef-bouillon (to which has been added 2 per cent. sugar, with, occasionally, the addition of starch,) urine, milk, watery extracts of various plants or grains, juice of fruits, saliva (to which some nutritious substances has been added), etc.

Pure cultures are obtained by transferring a minute quantity of a "colony" from a glass plate, or Petri dish, on the end of a sterilized platinum needle, to a tube of nutrient medium, where it is "planted" by either thrusting the needle directly through the centre of the solidified culture medium in a test-tube, then twisting the needle a few times between the thumb and fingers, and carefully withdrawing same, (the so-called "thrust," "puncture," or "stab" culture), or by drawing the point of the impregnated platinum needle, which has been slightly bent, across the *surface* of the medium, which has been allowed to solidify in the tubes, in an oblique direction (the so-called "scratch," or "surface" culture.)

Considerable work and investigation in this line can be done by the practicing dentist, or physician, without investing in an expensive outfit; *al least* it is quite practical for any practitioner, who desires to determine the existence of any particular species of micro-organisms in certain cases, to *himself* inoculate a prepared tube of gelatine, or agar, with the suspected material, and send the same immediately to a bacteriologist for further treatment, examination, etc. The following is a procedure I have employed in the investigations of this subject, and is, as you will admit, exceedingly simple and quite satisfactory. I use the glass phials of various sizes, used by some wholesale drug houses for holding physicians' samples of pills, parvules, tablets, etc. These phials are sterilized, filled with from 1 to 2 ctm. sterilized gelatine, or agar, and their mouths closed with the ordinary cotton-wool plug. When a patient comes under treatment, having a lesion of the mouth, teeth, or gums, the bacteria of which it is desired to cultivate, the dentist sterilizes an excavater, by passing it back and forth through the flame of his spirit lamp, or Bunsen burner, a few times, and carefully removes a bit of material from the mouth and pushes it just into the gelatine in the prepared bottle. The same day, at the first opportunity, this is sent to me, and dish-cultures, etc., are subsequently made from the specimens.

To review *all* the varieties of bacteria which have thus far been described and obtained (over 100 species) from the buccal cavity, with their individual peculiarities, etc., would require several papers, each fully as long as the present one. I shall, therefore, confine myself to the consideration of a few of the more prominent and frequently occuring varieties, and afterward offer some comments, applicable, in a *general* way, to the subject.

It has been customary for many observers, to classify every thread-producing germ, which they find in the buccal cavity, as "leptothrix buccalis." This is to be deprecated, as there are several bacteria of the mouth which form threads.

The name "leptothrix buccalis" (like "bacterium terms"), designates no particular organism, possessing peculiar characteristics, and the name no more deserves to be retained than "denticola," "Buhlmann's fibres," etc.; the less so since it has always been the expression for an obscure and erroneous conception. Morphologically, as well as physiologically considered, leptothrix buccalis has been regarded as a veritable wonder. It has been said to perforate and split up teeth, its elements to cause all kinds of diseases in the oral cavity, to penetrate into the lungs, the stomach, and other parts of the body, and everywhere to manifest a destructive influence.

As absolutely nothing was known concerning the biology and pathogenesis of this organism, all sorts of wonderful properties were ascribed to it. It is therefore high time to banish this confusing name from bacteriological writings. (Miller.)

Miller suggests the name of leptothrix innominata for those germs of thread-like growth, whose biology is too ill understood, to place their relation to other mouth-bacteria.

Let us, for a moment, consider what the inducements are which the mouth, as a whole, offers to wandering, homeless "bugs," that they so readily and promptly enter these premises, and not only obtain their own individual livelihood, but, unceremoniously, at once proceed to increase the members of their households.

As pertinently stated by one observer,* the mouth forms "a kind of hot-house, or forcing ground, for their cultivation." Dr. Bergtold† says: "If one could find a perfectly sterile mouth, he could also see at once that the opportunies of seeding it, so to speak, are excellent, in that every individual is more or less constantly taking in air and also food and drink through that channel, and in both

* Woodhead—"Bacteria and Their Products," p. 337.

† "The Mouth as a Center of Infection," W. H. Bergtold, M. D., Dental Cosmos, Vol. 43, No. 2.

these actions receiving numberless spores and other forms which, later, gives us growth of bacteria.

The organic and inorganic substances found in the mouth, which may serve as nutriment for micro-organisms, are the following:*

1. Normal saliva;
2. Buccal mucus;
3. Dead epithelium;
4. Dental tissue softened by acids;
5. Exposed pulps;
6. Exudations of the gums, conditioned by the irritation of tartar, etc.;
7. Accumulation of particles of food.

The carbohydrates and albuminous substances furnish the greatest nutriment for bacteria in the mouth, and are almost constantly present there at all times. They are found between the teeth, in cavities in the teeth, and also upon their surface, and in any depressions.

Perfectly pure, mixed, human saliva has no toxic properties, and in those cases which have been reported by Pasteur, Valpian, Gautier and others, in which "unadulterated human saliva caused more or less morbid phenomena," it must be suspected that the samples used *were*, in some manner, contaminated.

Mixed with the various deposits of bacteria, etc., always present in the mouth, saliva may possess most energetic toxic properties, having many times proved fatal, even as abundantly proved by numerous recorded cases.

It is also probable that the saliva has far less antiseptic properties than is often ascribed to it, and the undisturbed growth of micro-organisms in the oral cavity, would seem to sufficently support this view.

The diastatic action of the ptyalin of the saliva, in changing starch into a variety of sugar, variously termed

* "Micro-organism of the Human Mouth," Miller, p. 37.

dextrose, maltrose, or ptyalose, which, as soon as formed produces an excellent culture-medium for those germs concerned in the process of fermentation.

According to Miller,† there are six different micro-organisms which are almost invariably present in every mouth. They are:

1. *Leptothrix innominata.* This occurs as thin, more or less zig-zig threads. Found in the soft white deposit on teeth in *every* mouth. Is stained faint yellow by a solution of iodine in iodide of potassium sol., slightly acidulated with lactic acid.

2. *Bacillus buccalis maximus.* Occurs as isolated bacilli, or threads, more often as "tufts of threads." Is the largest of mouth bacteria. Is stained brownish-blue more or less deeply, with iodine solution.

3. *Leptothrix bucculis maxima.* This occurs as long, thick, straight or curved filaments, somewhat similar to bacillus buccalis maximus. Is found on the mucus deposit upon the teeth. Is not stained by the iodine solution.

4. *Jodococcus vaginatus.* Occurs singly or in chains of from four to ten cells. Chains have a sheath, and cells appear as flat disks, or as more rounded, even squares. Occurs in all unclean mouths. Not stained with the iodine sol.

5. *Spirillum sputigenum.* This is seen as rods, curved like commas, having very active, spiral movements. Found in all mouths, especially in unclean ones. Is the soft deposit on the margin of inflamed gums of dirty mouths. Stains more readily than the foregoing.

6. *Spirochaete dentium* (denticola. Found as spirals of very irregular windings and unequal thickness. It is found under the margins of the gums, when covered with a dirty deposit and slightly inflamed, or in other words, gingivitis marginalis.

† "Micro-organisms of the Human Mouth," Miller, p. 69.

There are a great many mouth bacteria proper, not *invariably* found in *every* mouth, which are uncultivable and whose pathogenesis is unknown. Among them Miller found a bacterium of enormous dimensions, in the mouth of a dog suffering from pyorrhœa alveolaris. which he has called leptothrix gigantea. There are also three or four kinds of germs from the mouth which give a blue, or violet reaction with iodine, and from twenty to forty varieties which are cultivable, partly non-pathogenic, partly of unknown pathogenesis. The oral cavity is a favorite locality for many varieties of chromo genic or color-producing bacteria. These are widely diffused in nature, and occur abundantly in water, in the air, and various places.

In the mouth they are less numerous than the colorless germs, and probably on account of this preponderance, the color is not visible when in this locality, but is readily developed during the culture of these micro-organisms on many of our culture media.

Among the colors produced by different species of the mouth bacteria, Miller, gives a ,yellow, produced by at least eight kinds of bacteria : *green*, (by 5), *red*, or reddish (by 3), *blue*, *black*, *brown*, *orange*, etc.

To study any of the bacteria, particles of matter containing them must be taken from the mouth, and mounted directly on covered glass, after which they may be examined fresh and unstained, or (after being carried through the flame of a Bunsen burner or spirit lamp *three times*) be stained and mounted permanently.

Many of the germs that have been found in the oral cavity are, of course, accidentally present, having been deposited just previous to an examination, which would only remain a short period.

The micro-organisms I have just mentioned, as being termed "Mouth Bacteria Proper" by Miller, are found in *all healthy* conditions of the cavity, but the variety and number is more or less greatly augmented when any morbid condition whatever occurs, such as inflamed gums, wounds, etc., of the mucous membrane of the mouth, dental caries and ulcerations, etc. Of these Miller found, by inoculation experiments on mice, rabbits and guinea pigs, many germs, the inoculation with which produced either death, or a pathological condition of the animals thus treated. These he calls "*Pathogenic Mouth Bacteria.*" Of the varieties separated, the following were studied more in detail :

1. Micrococcus gingivæ pyogenes;

2. Bacterium gingivæ pyogenes;

3. Bacillus dentalis viridans;

4. Bacillus pulpæ pyogenes.

The first of these micro-organisms, *mic. ging. pyog.*, was found several times in a case of pyorrhœa alveolaris, in the deposit around the teeth of a very filthy mouth.

The second, *bact. ging. pyog.* was found in the same mouth as the foregoing, and also in a suppurating tooth-pulp of a second person.

*Bact. dent. virid.*, the third variety, was found in the superficial layers of carious dentine. In cultivation upon gelatine this bacterium produces a beautiful opalescent green coloring matter which it imparts to the gelatine.

The fourth bacterium, *bact. pulp. pyog.*, was found in gangrenous tooth-pulp. The inoculating materils used, were pure cultures of the different germs, mixed cultures and gangrenous pulps, and the inoculations were made into pockets beneath the skin of the animals and by subcutaneous injections, and injections into the abdominal and thoracic cavities. The pockets were made as usual, at the root of the tail, and the injections with sterilized syringes.

Before giving the results of these inoculations, let us see what are the conditions necessary to be fulfilled, in order to establish unrefutable proof of the pathogenic nature of any given bacterium. According to Koch, a micro-organism must comply with the following requisitions, before its pathogenic character is determined, the so-called "*Koch's laws*, or *rules*:"

First—It must be proved to be present in all cases of the disease in question.

Second—It must further be present in this disease and in no other, since otherwise it could not produce a specific definite action.

Third—A specific micro-organism must occur in such quantities, and be so distributed within the tissues that all the symptoms of the disease may be clearly attributable to it.

Fourth—After removal from the body of an affected animal, and its growth in pure culture, the inoculation of the latter into susceptible animals, must produce the disease in question.

Miller's inoculation were followed by either local redness and swelling, abscesses, suppuration and gangrene of adjacent tissues, or blood poisoning, and in many cases by death from septicæmia peritonitis, pleuritis, etc.

Inoculations with mixed cultures proved more dangerous than when pure cultures were used, and still more effective when gangrenous pulps were used than with mixed cultures.

The diseases caused by the pathogenic bacteria of the mouth Miller considers under six heads, according to the point of entrance of the infection :

1. Infections caused by a breach in the continuity of the mucous membrane, brought about by mechanical injuries, (wounds, extractions, etc.). These lead, either to local or to general disturbances.

2. Infections through the medium of gangrenous tooth-pulps. These usually lead to the formation of abscesses at the point of infection (abscessus apicalis), but also sometimes to secondary septicæmia and pyæmia with fatal termination.

3. Disturbances conditioned by the resorption of poisonous waste products, formed by bacteria.

4. Pulmonary diseases caused by the inspiration of particles of slime, smell pieces of tartar, etc., containing bacteria.

5. Excessive fermentative processes, and other complaints of the digestive tract. caused by the continual swallowing of microbes and their poisonous products.

6. Infections of the intact soft tissues of the oral and pharyngeal cavities, whose power of resistance has been impaired by debilitating diseases, machanical irritations, etc.

7. Possible infections by the accumulations of the excitants of diphtheria, typhus, syphilis, tuberculosis, etc.

### DENTAL CARIES.

It has been proven, beyond doubt, that decay of the teeth is caused by two different processes, namely: (a) chemical, (b) parasitical.

The first is a decalcification of the enamel, or dentine, or both, caused by the presence of acids in the mouth, which have been formed from the fermentation of starch and saccharine substances, resulting in a softening of these tissues, after which these latter form an excellent food-medium for many varieties of bacteria.

The prevention of dental caries depends, first of all, on strict cleanliness of the mouth, the importance of which can not possibly be over-estimated, the details necessary for the proper fulfillment of the same would be superfluous for me to suggest to you. Undoubtedly, good stiff tooth brushes and plenty of clean water stand at the head of all measures of this nature. The next prophylactic means is the intelligent use of proper antiseptics.

By far the most perfect germicide known, that can be at all employed in this connection, is the bichloride of mercury, but the use of this substance is not without danger. It should not be used as a wash for the mouth in solutions of greater strength than 1 to 2,000, and even then care must be exercised in its application. Other antiseptics which have been recommended for the oral cavity are salicylic acid, strength of 1 : 200, or 1 : 350 listerine, wintergreen oil, and like aromatic substances are suggested.

In this connection might be noted the germicidal properties of tobacco, either the juice of the leaf or the smoke of the burning leaves. Certain it is, from results obtained by many experiments and observations, that tobacco-juice, or smoke, very speedily destroys bacterial life, but I would not, on this account, advocate its use, as the evil results of excessive indulgence in the "weed" more than counterbalance any possible benefits resulting from its antiseptic action on micro-organisms of the mouth.

In discussing the subject of infection, attention should be directed to the danger which exists from the spread of infectious forms of bacteria that are liable to be present in the mouth in various directions. It is not difficult, under certain circumstances, to excite an inflammatory process in the middle ear, the transmission of septic germs taking place through the eustachian tubes; similar results may also occur from pyogenic bacteria being carried from the mouth to the throat, lungs, parotid gland, antrum, and even to the brain, as stated by Bergtold.

When it is considered that of all diseases of a parasitic nature to which mankind is susceptible, dental caries is by far the most frequent, the possibilities, I have just mentioned, can not be charged as being the improbable and unlikely speculation set forth by one who is "cranky" on the subject.

Upon reviewing the various literature on this question, especially those portions of it which refer to the dangers of infection between the dentist and his patient, the speaker was much surprised to find no advice offered to the dental profession by competent bacteriologists, as to the considerable (and oft-times *great*) danger present, to the patient, by pathological conditions the dentist *himself* may be suffering from at the time of operating, and to point out the necessity of establishing, by legislative measures if required, laws, or statutes, which would prevent the occurrence of such dangers.

I refer more particularly to the jeopardy in which human life is placed, when people are subjected to treatment by a practicing, *tubercular* dentist. This may seem, to many of you, as a bit of superfluous advice, and you may retort that such a circumstance is beyond the bounds of possibility, but I assure you, *I have seen a tubercular member of your profession practicing daily on unsuspecting, or ignorant, and innocent patients.*

My experience with dentists has been very limited, and therefore, I am not in a position to make any assertions as to the frequency of such pernicious and dangerous proceedings; but the very fact that my slight personal observations resulted in the detection of *one* such case naturally sugges s a possible more common occurrence of tuberculosis in practicing dentists than might be supposed. Since commencing the work incident to the preparation of this paper, one of the local members of your profession has detailed another case, which was under his own personal observation, of a "TUBERCULAR PRACTICING DENTIST."

The *greatest* danger, under such circumstances, *is not,* as some of you might imagine, in the infection of the patient by the transmission of the germs through the medium of the breath of the operator, but in the reception of tubercular material, which becomes dry on the handkerchief, clothing, linen, or instruments of the dentist. The prevention of such desiccation is so extremely difficult and impracticable as to be discarded without serious consideration, if such (prevention) be presented as a possible prophylactic measure, to enable the victim of this malady to continue his professional work until physically unable to do so on account of the inroads of the disease.

It is not generally known that bacteria do not float in the atmosphere in the moist state, but only do so after desiccation, and then probably to no great extent, unless aided by more or less strong currents of air.

Tuberculosis is now almost universally considered to be an infectious disease, and of so contagious a nature that

I candidly believe, we shall, many of us, see the day when attention to preventive measures against possible infection from cases of this disease is as regularly insisted upon as are the sanitary requirements in cases of small-pox, yellow fever and typhus fever (with the exception of somewhat less vigorous quarantining) at the present day. The period in which to accomplish this much desired treatment of tubercular cases will depend upon the rapidity with which the laity, and *professional men* even, become educated to the full comprehension of the single and *sole* cause of the affection, the tubercle bacillus, and the proper realization of the benefits to be derived from the adoption of such measures. And to the intelligent efforts and advice of the members of the medical profession, as well as to the great aid which *you*, members of the dental profession can give by embracing each and every opportunity to inform your patients, especially influential citizens, as to the true character of tuberculosis, must the accomplishment of this end very largely devolve.

Of all the various ways by which tubercle bacilli find entrance into the human body (such as from the surface of the skin through wounds, by contusions, cuts, or otherwise; from the ingestion of milk and flesh from tubercular cows and animals, etc.), infection by inspiration—by the entrance of the dried germs through the mouth, and so on to the lungs—far surpasses in frequency all other methods of transmission. And this can only be accomplished when the medium on which the micro-organisms have been discharged from the body dries, or disintegrates into powder or dust. For this reason the most dangerous source of infection is from handkerchiefs, or cloths on which the sputum has been received (unfortunately a too common procedure) and on which it becomes dry in an exceedingly short time. Consequently by merely preventing the spucum of phthisical persons from drying the most important kind of infectious matter may be rendered harmless.

The first contradiction I have seen by competent pathologists (who acknowledge the tubercle bacillus as the essential cause of tuberculosis) to the statement that the *commonest* source of infection is the inhalation of dried bacilli from handkerchiefs, linen, clothing, dust from the floors and ceilings of rooms previously occupied by tubercular subjects, etc., was recently made by Dr. J. West Roosevelt, of New York.*

Dr. Roosevelt maintained "that there was much more likelihood of getting an over-dose of the virulent germs" (of tuberculosis) "through the alimentary tract, either by the ingestion of meat, milk, or in children, by putting articles of every nature into their mouths, no matter where they have lain," than by the manner which I have just described. While recognizing the value of the opinion of so able an authority as Dr. Roosevelt, I am convinced that his statement will not be corroborated by the majority of pathologists and bacteriologists in this country, or on the continent. True it is that much needless alarm may be created in the minds of the public by the advocacy of too severe and unnecessary measures of prevention, such as quarantining, etc, by prejudiced and over-zealous investigators, the effcct of which would be the unjustifiable persecution of many poor victims of tuberculosis, and as I just suggested, the *only proper* course to pursue in dealing with this question is by persistently and intelligently educating the minds of the publib as to the *exact* status of this matter.

Few pathologists at the present day believe in the theory of heredity as a cause of tuberculosis. But it is a fact that the off-spring of consumptive parents, especially where the mother is affected, often develop sooner or later the disease, but the explanation is to be sought in a general impoverished condition of the organism, as in-

---

* New York Academy of Medicine, Feb. 4, 1892; vide Medical Record, Vol. 41, No. 10.

dicated by enfeebled assimilative and nutrient powers, which are quite sure to follow as the inheritance from unhealthy progenitors.

What I have just said in regard to transmission naturally leads to the most interesting and important problem in bacteriology, namely, that of *immunity*; and I ask your indulgence for a few moments in order to state the theories at this time held in regard to this subject.

As you are probably all aware two principal views are advanced to account for the difference of susceptibility possessed by different animals to the same micro-organism, and also that exhibited by the *same* animal to *different* germs.

I might state here that immunity is of two principal kinds, to wit: (a) *natural*, or "in-born;" (b) *acquired*, or "artificial."

The first of the theories of *natural* immunity is based on the *chemical* germicide properties of the blood-serum and tissue-juices of the body.

The second, or "*Metschnikoff's*" theory attributes the resisting power which an individual or animal may possess to the so-called "*phagocytic*" action of the tissue-cells of the body, more especially the colorless corpuscles, or leucocytes of the blood.

Metschnikoff believes the presence or absence of immunity depends on the ability or inability of the cells of the body to devour and destroy the bacteria. Such ability may be natural or acquired. In the latter case the cells, where they have once had the opportunity of devouring attenuated micro-organisms with a milder poison, which nature enables them to withstand, are so far accustomed to it that they can devour the most virulent material with impunity. This can be effected both by gradual adaptation, and also by a kind of selection in which only the strongest, most vigorous cells remain and transmit the acquired faculty to their descendants. The leucocytes are but short-lived cells. A permanent resistance of the organism to a disease which it has once had, or against which it has been protected by inoculation is, therefore, only conceivable if we grant to the cells the power of transmitting an acquired property unaltered to their children and their children's children. This hypothesis, as must have been seen, presupposes an extraordinary docility in the protoplasm of the white blood-corpuscles, to which it attributes something like feeling, thinking and acting, a sort of mental perception. But if we raise no objection to this, there remain plenty of reasons for combating the phagocytic theory as immunity.

In our opinion, the fact that it is essentially the excretions of the bacteria whieh produce, or are able to produce immunity, is difficult to harmonize with Metschnikoff's hypothesis, for if no living micro-organisms are present none can be devoured to accustom the cells to the poison and prepare the way for resisting more virulent successors To overcome this difficulty it would be necessary to suppose that the reception of attenuated germs acts upon the cells only as a specific stimulant, to which they answer by a functional reaction, and that this stimulating power exists in the same degree, and works in the same manner, also, in the bacterial products. The theory of the germicidal action of the blood-serum, or plasma is, I believe, supported by more weighty authority than is the ingenious one of Metschnikoff.

I have thus forsaken the exact theme of my discourse from a belief that more benefit would be derived from the brief treatment of a subject, at best only partly understood by the highest authorities in this branch, but which one who has devoted much time in practical investigations must, necessarily, be better qualified to handle than the average practitioner.

Would the time (and, perhaps, your forbearance also) admit, much profit might be had from the further discussion of bacteria of the mouth, but the vast extent of the subject renders anything but a superficial consideration of it impossible.

As yet, we are only on the *frontier* of the domain of bacteriology, and have only obtained a comparatively few facts, or details, of this most interesting and important kingdom.

What data and facts, investigations into the interior of this boundless area of unexplored territory of micro-organic life, will place us in possession of, time, perseverance and unremitting efforts will prove.

We certainly have reason to believe that the knowledge of such points, at present undiscovered, together with their practical application, will be of inestimable value to mankind.

In closing, allow me to express my gratification for your kind invitation to participate in the proceedings of your valuable and flourishing society, and to thank you sincerely for the close and courteous attention accorded to me to-day, and further, to record my obligations to one of your individual members, Dr. Hodge, who has rendered me every assistance possible in the collection of material for and in the preparation of this paper.—*Microcosm.*

